# German medical students’ learning environment during the fading COVID-19 pandemic

**DOI:** 10.1186/s12909-026-10827-1

**Published:** 2026-11-11

**Authors:** Marvik Leich, Jennifer Guse, Corinna Bergelt

**Affiliations:** 1 Department of Medical Psychology, University Medicine Greifswald, Greifswald, Germany; 2 Department of Psychiatry and Psychotherapy, Clinical Division of General Psychiatry, Medical University of Vienna, Vienna, Austria; 3 Department of Medical Psychology, University Medical Center Hamburg-Eppendorf, Hamburg, Germany

**Keywords:** Learning environment, Study motivation, COVID-19, Loneliness, Depression, Anxiety, Mental health

## Abstract

**Background:**

The Medical School’s learning environment (MSLE) is important for medical students’ academic performance and mental well-being. COVID-19 disrupted medical education worldwide through restrictions on practical and in-person teaching. Although several studies have showcased the immediate effects on medical students’ perceptions of MSLE and mental well-being, less is known about the changes during the transition to post-pandemic medical education.

**Methods:**

Four cross-sectional online surveys were conducted at a German medical school between winter 2021/2022 and summer 2023. MSLE was assessed using three items from the Johns Hopkins Learning Environment Scale (JHLES) and three self-developed items. Various validated items assessed distress (DT), perceived stress (PSS-4), depression (PHQ-2), anxiety (GAD-2), and loneliness (R-UCLA-3). Analyses included group comparisons and binary logistic regression.

**Results:**

Across four survey waves, 687 individual medical and dental students participated. Positive MSLE perceptions were more frequent in later waves, although differences were not significant after correction for multiple comparisons. Social connectedness and access to mentoring were reported significantly more frequently in later waves. Higher age, more advanced study years, greater perceived stress, and perceived lack of practical experience were associated with lower odds of positive MSLE. The proportion reporting decreased study motivation since the onset of COVID-19 was significantly lower in the final wave. Older students, those in more advanced study years, and those experiencing loneliness or distress more frequently reported decreased motivation. Mental burden showed varying patterns: loneliness was generally lower in later waves, distress peaked in summer 2022, depressive symptoms and perceived stress in winter 2022/2023, and anxiety in summer 2023. After correction for multiple comparisons, only differences in distress across survey waves remained significant. Female students reported higher anxiety, loneliness, and perceived stress than male students, but only the gender difference in anxiety remained significant after correction.

**Conclusion:**

Medical students reported a more positive perception of the MSLE in later survey waves during the transition from pandemic to post-pandemic medical education. However, these improvements were not matched by a uniform recovery in medical students’ well-being. Medical schools are therefore encouraged to strengthen students’ learning environments by ensuring learning opportunities, mentoring, and mental health support throughout training.

**Supplementary Information:**

The online version contains supplementary material available at https://doi.org/10.1186/s12909-026-10827-1.

## Introduction

Learning is an active process involving changes in knowledge, beliefs, behaviors, or attitudes, which students achieve rather than passively receive in an academic context [1]. Theoretical constructs of learning contexts, or learning environments (LE), vary greatly depending on the definition. One commonly used approach describes the learning environment in medical school as the social, physiological, and physical context in which learning occurs [2, 3]. Although multiple definitions coexist, the overall importance of LE on students’ various domains of well-being is unchallenged. For one, learning climate strongly determines students’ behavior [4]. Better perceived LE has been shown to be positively associated with better academic performance [5, 6], higher quality of life [7–10], better emotional well-being [11, 12], as well as less psychological distress [8, 13] and lower odds of symptoms of burnout, like emotional exhaustion and depersonalization [7, 13].

While gaining insight into medical students’ learning experiences, one cannot overlook the impact of the COVID-19 pandemic, which marked a major turning point in medical education in recent years. In response to the pandemic, medical schools implemented swift actions that disrupted face-to-face medical teaching and training [14], medical licensing exams [15], and clinical rotations [16]. These disruptions led to uncertainties among medical students regarding their future residencies [16] and career plans [14, 17]. Consequently, both medical teachers and students were forced to adapt to new forms of medical education, such as blended, asynchronous, and e-learning [18, 19]. At Greifswald University Medical School, teaching was predominantly conducted in person before the pandemic. From summer 2020 onward, most teaching shifted to digital and distance learning formats, while practical and bedside teaching was gradually reintroduced as pandemic-related restrictions eased. During the initial phase of COVID-19, students reportedly expressed mixed perceptions of this shift. Some medical students responded positively to new learning approaches such as blended learning [20, 21] and overall online learning [22], while others felt demotivated by online lectures [23, 24]. A large meta-analysis supports the conclusion that healthcare students prefer in-person teaching over asynchronous, single-user online teaching [14]. When focusing on students’ perception of LE in particular, several studies imply that the positive effects of the altered academic climate outweighed the negative effects in the early response to COVID-19.

Initial research showed that undergraduate medical students reported both higher levels of LE [21, 25, 26] and increased overall motivation [23], especially due to a sense of duty, altruism, and values associated with aspiring professionalism [27], particularly as many students gained experience as frontline healthcare workers [15]. Lower study motivation was reported by students with greater fear of COVID-19 [28], psychological distress due to the pandemic [20], or symptoms of burnout [29]. Additionally, students experiencing lockdown measures demonstrated significantly higher levels of amotivation [30]. Analyses further indicate a correlation between a lower perceived quality of the LE and symptoms of depression among undergraduate students [31]. With the transition to post-pandemic life up to 2023, data on students’ perceptions of their LE remain scarce. This study aims to address this research gap by evaluating perceived LE, study motivation, and mental well-being among German undergraduate medical students in 2022 and 2023.

## Materials and methods

### Study design

To assess LE and mental well-being among German medical students, this observational, cross-sectional study was conducted at a medical school in Germany (Greifswald University Medical School, UMG) over the course of 1.5 years (winter 2021/2022 to summer 2023). The study consisted of four individual surveys conducted at the end of each term, comprising four different cohorts: the winter 2021/2022 cohort (February–March 2022, referred to as t1), the summer 2022 cohort (July–September 2022, or t2), the winter 2022/2023 cohort (February–March 2023, or t3), and the summer 2023 cohort (July–September 2023, or t4). Both medical and dental students were invited to participate in four online surveys. Participation in one survey wave did not require participation in subsequent waves, and the composition of the four cohorts therefore differed. Thus, comparisons between survey waves reflect differences at the cross-sectional cohort level rather than within-person changes. SARS-CoV-2 dynamics changed rapidly during this study. In winter 2021/2022, the highest weekly peak in SARS-CoV-2 infections was registered in Germany [32]. By April 2023 - in line with the WHO’s declaration of the end of the COVID-19 global health emergency [33] - most COVID-19-related governmental regulations in Germany were abolished [34].

### Study setting and medical curriculum

The medical curriculum at Greifswald University Medical School is structured into preclinical and clinical study sections, followed by a final year of practical training [35]. During the preclinical section, students primarily learn the basic natural sciences and complete the First Medical Licensing Examination. The subsequent three-year clinical section covers theoretical and practical training in all major medical disciplines. At the end of the fifth year, students take the Second Medical Licensing Examination, followed by a practical year in which they complete supervised, full-time clinical rotations across different medical specialties and hospital departments.

Medical and dental students at Greifswald University Medical School share some parts of the curriculum but follow increasingly specialized clinical training in later stages of their studies. Before the COVID-19 pandemic, teaching was delivered predominantly in person and synchronously, while digital and asynchronous formats played only a minor supplementary role. At the beginning of the COVID-19 pandemic in 2020, medical teaching in total was initially suspended for approximately one month. From summer 2020 onward, most in-person teaching was replaced by digital and distance formats, including synchronous remote teaching and asynchronous online courses delivered through learning platforms. Moreover, all examinations were conducted online [36]. Teaching formats gradually returned to in-person delivery as COVID-19-related restrictions eased over the study period, in accordance with federal and state regulations. Priority was given to teaching activities that required direct interpersonal contact, such as bedside teaching and training for clinical examinations. In contrast, most lectures were held online until the end of all COVID-related regulations [37].

### Participants

All medical and dental students enrolled at UMG were invited to participate in the study (1,344 students in winter 2021/2022 and 1,294 students in summer 2023). Information about the study was distributed via emails from the Dean of Students and the Medical Students’ Union of UMG, as well as through social media posts. Additional posters promoting the study were displayed in central student areas, including the central clerk room and skills lab. Students were contacted at the beginning of the lecture-free periods in February and July, and reminders were sent during the survey periods. Participation was voluntary, and no incentives were offered. A total of 10 questionnaires from non-binary participants were removed from analyses to ensure respondents’ anonymity. Students were generally required to complete all questionnaire items, except one item assessing changes in study motivation since the beginning of the COVID-19 pandemic in the summer 2023 cohort.

This exception was made because students who had started medical school after the onset of the pandemic could not compare their current study motivation with a meaningful, pandemic-related baseline.

### Measures

First, participating students reported basic sociodemographic characteristics, including age, field of study, gender, current semester, and relationship status. Second, the learning environment, including study motivation, was measured using both established and self-constructed items. Lastly, validated instruments were used to assess overall psychological distress, self-perceived stress, loneliness, anxiety, and depressive symptoms. An English-language version of the questionnaire, including the respective sources of previously published scales and measures, is provided as Supplement File 3.

#### Learning environment

Participants’ overall perception of the Medical School Learning Environment (i.e., MSLE) was assessed by a five-point Likert scale item, with answers ranging from “terrible” and “poor” to “fair”, “good,” and “exceptional”. To allow further analyses, we categorized the overall perception of LE as “positive” or “not positive,” with both answers “good” and “exceptional” indicating a positive perception of LE. Furthermore, we selected three items from the widely used and validated Johns Hopkins Learning Environment Scale (JHLES) covering three domains of learning environment: community of peers (“How connected do you feel to other UMG students?”), academic climate (“UMG’s curriculum allows me to use my preferred learning style.”), and mentoring (“I have found a mentor in a clinical specialty or discipline that I am passionate about.”). All three items were Likert-scaled (1 = “strongly disagree” to 5 = “strongly agree”). For exploratory analyses, the three items were combined into a study-specific composite score to provide an additional, descriptive summary of these aspects of the learning environment. However, this composite score does not constitute an established JHLES subscale. Previous studies have demonstrated the validity of the original JHLES [38, 39]. Validation in the German population remains lacking.

To reflect on COVID-19–related study burdens, three self-developed items were used. First, changes in study motivation since the onset of the COVID-19 pandemic were assessed using a Likert-scale item (1 = “considerably improved” to 5 = “considerably worsened”). Based on the conceptual aim of providing a clear overview of the direction of motivational change and facilitating subsequent group comparisons, responses were dichotomized into “decreased” and “not decreased”. In addition, students rated how burdensome two specific aspects of their study situation had been over the past four weeks: preparation for examinations, and the loss or reduction of practical teaching (e.g., bedside teaching, clinical rotations, and practical courses).

#### Distress and self-perceived stress

To assess overall psychosocial distress, we applied the German version of the Distress Thermometer (DT), originally developed by the National Comprehensive Cancer Network (NCCN) for screening in cancer patients [40]. The DT comprises a single-item scale ranging from 0 to 10, with higher values indicating greater levels of distress. The NCCN guidelines recommend a cut-off score of ≥ 5 to identify individuals experiencing clinically relevant distress [41]. In samples of German cancer patients [42], the DT has demonstrated good validity. Although no studies have assessed the psychometric properties of this instrument in medical students, the DT has been shown to be a useful and highly accessible screening tool for health researchers [43]. Students’ self-perceived stress was measured using the four-item Perceived Stress Scale (PSS-4), a widely used and validated instrument [44, 45]. The PSS-4 assesses the frequency of stress-related events in the last month across 4 items, each rated on a 5-point Likert scale (0 = “never” to 4 = “very often”). Again, higher sum scores indicate greater perceived stress.

#### Depression and anxiety

Symptoms of depression and anxiety were evaluated using the German version of the widely used Patient Health Questionnaire-4 (PHQ-4) [46]. Originating from the more comprehensive Patient Health Questionnaire (PHQ-D), the ultra-short PHQ-4 integrates two items assessing depressive symptoms (PHQ-2) and two items assessing generalized anxiety symptoms (GAD-2). The PHQ-4 assesses self-perceived impairment due to depressive or anxiety symptoms over the last 2 weeks (0 = not at all, 3 = almost every day). Sum scores above 2 points were used as a cut-off to identify clinically relevant depressive or anxiety symptoms. Psychometric evaluation for the German general population has proven its reliability and validity [47].

#### Loneliness

Loneliness was evaluated using the ultrashort three-item version of the Revised UCLA Loneliness Scale (R-UCLA). This three-item form assesses interpersonal connectedness, social connectedness, and self-perceived isolation as key aspects of loneliness. Items were rated on a three-point Likert scale ranging from 1 (hardly ever) to 3 (often). In previous research, scores on the scale have been dichotomized into “not lonely” (sum scores 3–5) and “lonely” (sum scores 6–9). In US populations, the ultrashort UCLA 3-item scale has shown both satisfactory reliability and validity [48]. For German-speaking samples, sufficient and good psychometric validation is currently available only for the original 20-item Revised UCLA Loneliness Scale [49].

### Statistical analyses

Analyses were conducted using all available responses for each respective variable. Participants with missing values for a given variable were excluded only from analyses involving that particular variable. Descriptive statistics were used to summarize and characterize sample characteristics.

Thereafter, depending on the level of measurement and inclusion of covariates, group differences between survey cohorts were examined using chi-square tests, univariate analyses of variance (ANOVAs), and univariate analyses of covariance (ANCOVAs). Reported p-values are unadjusted. To control for an increased risk of type I errors due to multiple testing, Holm–Bonferroni corrections were applied within each table, with all comparisons within a given table constituting one family of hypotheses [50]. Lastly, associations with a positive perception of the learning environment were examined using binary logistic regression. Variables were included based on their conceptual relevance to students’ perception of the learning environment and/or because previous studies had identified them as potentially associated with differences in learning environment perceptions (i.e., gender and relationship status [51], study year [52], and study subject [53]). Without prior statistical selection based on significance or model fit, available sociodemographic, psychosocial, and mental health variables considered relevant were entered simultaneously into the model (i.e., forced-entry). Multicollinearity among predictors included in the regression model was assessed using variance inflation factors (VIFs) and tolerance values obtained from an auxiliary linear regression model. Finally, to account for repeated participation across survey waves, sensitivity analyses were conducted using generalized estimating equations (GEE), with participant ID as the clustering variable. For binary outcomes, logistic GEE models were used, while linear GEE models were applied to continuous outcomes. All statistical analyses were performed using IBM SPSS Statistics (version 31).

## Results

### Study sample

Across all four cohorts, 687 individual medical students participated in at least one survey. Of these, 538 participated in only one survey, 103 in two, 26 in three, and 20 in all four survey waves. Hence, response rates ranged from 12.0% (summer 2023) to 21.1% (winter 2021/2022) among all eligible students in each cohort.

Of the participating students, 606 (88.2%) studied human medicine and 81 (11.8%) dentistry. Sensitivity analyses of the main findings restricted to medical students showed that including dental students did not substantially affect the main findings (data not shown). The proportions of undergraduate students in preclinical and clinical training were nearly equal, except for the summer 2023 cohort (69.0% in preclinical training). 72.5% of participants were female. Most students were between 21 and 25 years old (57.6%) and were in a relationship (51.4%). The study samples did not differ significantly by gender, but did differ significantly by age (Table 1).

**Table 1:** Students’ characteristics

	winter 2021/2022	summer 2022	winter 2022/2023	summer 2023	
	*n* = 283	*n* = 231	*n* = 233	*n* = 155	
Sex	*%*	*%*	*%*	*%*	*χ² (p)*
* Male*	28.3	28.6	26.6	25.8	0.533 (0.912)
* Female*	71.7	71.4	73.4	74.2	
Age	M = 23.3 (SD = 3.2)	M = 23.8 (SD = 3.4)	M = 22.7 (SD = 3.4)	M = 22.4 (SD = 3.0)	
* ≤ 20 years*	19.1	13.4	27.9	31.6	27.651 ( **0.001**)
* 21–25 years*	59.0	61.9	53.2	55.5	
* ≥ 26 years*	21.9	24.7	18.9	12.9	
Study Year	n (%)	n (%)	n (%)	n (%)	
* Year 1*	75 (26.5)	54 (23.4)	74 (31.8)	62 (40.0)	24.967 (**0.003**)
* Year 2*	67 (23.7)	58 (25.1)	54 (23.2)	45 (29.0)	
* Year 3*	64 (22.6)	47 (20.3)	45 (19.3)	27 (17.4)	
* Year 4 +*	77 (27.2)	77 (31.2)	60 (25.8)	21 (13.5)	

Annotations: *n* Sample size, % Percentages, *M* Mean value, *SD* Standard deviation. χ² = chi-square-value. Bold p-value= significant after Holm-Bonferroni correction within the set of comparisons reported in this table.​ Response rate in each cohort: 21.1% winter 2021/2022, 17.6% summer 2022, 17.8% winter 2022/2023, and 12.0% summer 2023

### Medical students’ learning environment and factors associated with positive LE

Across survey waves from winter 2021/2022 to summer 2023, students reported significantly higher levels of connectedness with other students and were more likely to report having found a mentor in a clinical specialty or discipline. In contrast, the perceived opportunity to implement students’ preferred learning style did not differ significantly across survey waves.

In line with these findings, the exploratory three-item composite score of UMG students’ learning environment was significantly higher in later survey waves, with the lowest mean at the end of the summer term 2022 and the highest mean at the end of the summer term 2023. Furthermore, the composite score differed by study year, with first-year students reporting higher composite scores overall than students in more advanced study years. However, we observed the largest difference in the composite score among fourth- and higher-year students from the winter 2021/2022 to the summer 2023 cohort. Composite scores among second- and third-year students did not differ significantly across survey waves. Differences in mean scores between female and male students were not statistically significant. All associated effect sizes were consistently small (Table 2).

**Table 2:** JHLES-based Learning Environment measures between winter 2021/2022 to summer 2023

	in total	winter 2021/2022	summer 2022	winter 2022/2023	summer 2023			
		*n* = 283	*n* = 231	*n* = 233	*n* = 155			
	M (SD)	M (SD)	M (SD)	M (SD)	M (SD)	SQ (MS)	F-value	*p*-value (η²)
Exploratory composite score	8.0 (2.3)	7.9 (2.2)	7.8 (2.3)	8.2 (2.3)	8.5 (2.7)	61.215 (20.405)	4.002	**0.008** (0.013)
* Community of peers*	3.3 (1.1)	3.1 (1.1)	3.3 (1.2)	3.5 (1.0)	3.5 (1.1)	27.625 (9.208)	7.778	**< 0.001** (0.025)
* Academic climate*	2.7 (1.1)	2.8 (1.1)	2.6 (1.1)	2.9 (1.1)	2.7 (1.8)	6.182 (2.061)	1.744	0.156 (0.006)
* Mentoring*	2.0 (1.1)	1.9 (1.0)	2.0 (1.1)	2.0 (1.1)	2.3 (1.2)	14.360 (4.757)	4.085	**0.007** (0.013)
Study year								
* Study year 1*	8.4 (2.3)	7.9 (2.3)	8.6 (2.6)	8.2 (2.1)	9.1 (2.0)	44.096 (14.699)	2.883	0.035 (0.010)
* Study year 2*	7.9 (2.3)	7.8 (2.1)	7.7 (2.1)	8.3 (2.4)	7.8 (2.5)			
* Study year 3*	7.9 (2.1)	8.1 (2.3)	7.4 (2.1)	7.9 (2.0)	7.9 (1.7)			
* Study year 4+*	7.9 (2.4)	7.7 (2.2)	7.6 (2.3)	8.2 (2.7)	9.3 (2.6)			
Gender								
* Female*	8.0 (2.2)	7.8 (2.1)	7.8 (2.4)	8.1 (2.2)	8.3 (2.2)	19.014 (19.014)	3.729	0.054 (0.004)
* Male*	8.2 (2.4)	7.9 (2.4)	7.8 (2.2)	8.5 (2.6)	9.1 (2.5)			
Study subject								
* Human medicine*	8.0 (2.2)	7.8 (2.2)	7.8 (2.2)	8.0 (2.2)	8.5 (2.2)	42.240 (21.210)	4.142	0.016 (0.009)
* Dentistry*	8.7 (2.6)	8.4 (2.2)	8.3 (3.1)	9.3 (2.6)	8.3 (2.9)			

Annotations: *n* Sample size, *M* Mean value, *SD* Standard deviation, *SQ* Sum of squares, *MS* Mean square, *F* F-value, η² Eta-squared effect size, *df* Degrees of freedom. Bold p-value= significant after Holm-Bonferroni correction within the set of comparisons reported in this table. Exploratory composite score = composite sum-score of the three Johns Hopkins Learning Environment Scale (JHLES) items

Over all time points, almost 1 in 2 students (48.6%) rated their MSLE as good or excellent (i.e., positive MSLE). The proportion of students reporting a positive MSLE differed from 44.9% in the winter 2021/2022 to 54.9% in the winter 2022/2023 cohort, although the differences across survey waves did not remain statistically significant after Holm–Bonferroni correction. Students under 20 and first-year students reported a positive perception of MSLE significantly more frequently than older and more advanced students. Post hoc analyses showed that pre-clinical students more frequently reported a positive perception of the learning environment than clinical students. After Holm–Bonferroni correction, only the overall group difference remained statistically significant (Supplement File 2). As older age does not necessarily correspond to a more advanced academic year, an additional subgroup analysis was performed among students aged 26 years or older. In contrast to the findings observed in the overall sample, no significant association between academic year and a positive perception of the learning environment was found at any of the four survey waves. However, subgroup sizes were very small, and these findings should therefore be interpreted with caution (data not shown). Additionally, students experiencing clinically relevant levels of depression, loneliness, and distress reported a positive MSLE significantly less frequently. All associated effect sizes were consistently small (Table 3).

**Table 3:** Change of medical students’ satisfaction with Learning Environment in fading COVID-19 pandemic (2022 to 2023)

		in total	winter 2021/ 2022	summer 2022	winter 2022/ 2023	summer 2023		
			*n* = 283	*n* = 231	*n* = 233	*n* = 155		
		%	%	%	%	%	χ²	*p*-value (V)
Overall perception of positive MSLE		48.6	44.9	44.2	54.9	52.3	7.971	0.047 (0.094)
Age	*< 20 years*	62.3	55.6	67.7	67.7	59.2	33.309	**< 0.001** (0.192)
	*21–25 years*	48.8	44.3	42.7	58.1	54.7		
	*> 25 years*	32.8	37.1	35.1	27.3	25.0		
Gender	*female*	49.2	45.8	46.1	53.8	53.0	0.436	0.509 (0.022)
	*male*	46.8	42.5	39.4	58.1	50.0		
Study year	*Study year 1*	61.1	57.3	61.1	67.6	58.1	30.865	**< 0.001** (0.185)
	*Study year 2*	48.7	44.8	44.8	53.7	53.3		
	*Study year 3*	45.4	46.9	36.2	53.3	44.4		
	*Study year 4+*	36.5	31.2	36.1	41.7	42.9		
Study subject	*medicine*	47.8	43.8	44.4	52.9	52.8	1.637	0.201 (0.043)
	*dentistry*	54.8	55.6	41.7	69.0	46.2		
Variables of mental burden								
Depression	*< cut-off*	56.3	58.0	48.6	59.1	59.0	23.424	**< 0.001** (0.161)
	*> cut-off*	40.1	27.3	40.0	50.8	44.4		
Anxiety	*< cut-off*	53.6	50.6	50.9	55.2	63.8	7.873	**0.005** (0.093)
	*> cut-off*	44.2	37.8	38.4	54.7	45.4		
Loneliness	*< cut-off*	55.7	52.0	51.2	60.9	60.0	20.329	**< 0.001** (0.150)
	*> cut-off*	40.7	39.1	35.6	47.0	42.9		
Distress	*< cut-off*	56.3	48.5	50.8	64.6	66.0	10.031	**0.002** (0.105)
	*> cut-off*	45.0	42.9	41.9	50.0	46.3		

Annotations: *n* Sample size. Overall perception of positive MSLE = response “good” or “excellent”. % = percentage of students reporting a good or excellent learning environment. χ² = chi-square-value. *V* Cramer’s V, effect size measure for chi-square tests. Bold p-value= significant after Holm-Bonferroni correction within the set of comparisons reported in this table. Distress = DT score > 4 points, Depression = PHQ-2 > 2 points, Anxiety = GAD-2 > 2 points, Loneliness = UCLA-3 > 5 points

To identify factors associated with a positive MSLE, a binary regression analysis was conducted, explaining 20.6% of the variance in total. With VIF values ranging from 1.024 to 2.475 and tolerance values ranging from 0.404 to 0.976, the predictors showed no evidence of substantial multicollinearity based on commonly applied thresholds [54]. In this model, higher age was significantly associated with lower odds of reporting a positive MSLE (OR = 0.90 per year). Similarly, students in clinical training (study year 3: OR = 0.51; study year 4+: OR = 0.34) had significantly lower odds of reporting a positive MSLE than students in year 1. Given the differences in sample composition across survey waves, particularly in summer 2023, an additional analysis adjusting for age and academic year was conducted. This analysis showed an attenuation of the association between survey period and positive MSLE perception (unadjusted: *p* = .047; adjusted: *p* = .275; data not shown).

Among mental health variables, higher self-perceived stress (OR = 0.88) was significantly associated with lower odds of reporting a positive MSLE. A greater lack of practical experience (OR = 0.76) was also significantly associated with lower odds of a positive MSLE. All other variables included in the model (i.e., gender, study subject, relationship status, distress, depression, anxiety, loneliness, and burden of exam preparation) were not significantly associated with a positive perception of the MSLE (Table 4). The main associations remained robust in sensitivity analyses accounting for repeated observations using generalized estimating equations (GEE). (Supplement File 4).

**Table 4:** Factors associated with positive LE in the fading COVID-19 pandemic: forced-entry binary logistic regression

						95% CI for OR	
		B	SE	OR	*p*-value	Lower CI	Upper CI
Sociodemographic characteristics							
Participation (winter 2021/2022*)	*summer 2022*	-0.078	0.201	0.925	0.699	0.624	1.372
	*winter 2022/2023*	0.173	0.204	1.189	0.396	0.797	1.774
	*summer 2023*	-0.008	0.224	0.992	0.970	0.639	1.539
Age	*continuous*	-0.106	0.027	0.899	**< 0.001**	0.852	0.949
Gender (female*)	*male*	-0.075	0.166	0.928	0.651	0.670	1.284
Study Subject (human medicine*)	*dentistry*	0.283	0.243	1.326	0.245	0.824	2.137
Study Year (Year 1*)	*Year 2*	-0.366	0.206	0.693	0.076	0.463	1.039
	*Year 3*	-0.668	0.228	0.512	**0.003**	0.328	0.801
	*Year 4 +*	-1.087	0.267	0.337	**< 0.001**	0.200	0.569
Relationship (not single*)	*single*	0.109	0.151	1.116	0.469	0.830	1.500
Variables of mental burden							
Distress (< cut-off*)	*> cut-off*	0.075	0.192	1.078	0.697	0.739	1.571
Depression (< cut-off*)	*> cut-off*	-0.339	0.204	0.712	0.096	0.478	1.062
Anxiety (< cut-off*)	*> cut-off*	0.158	0.206	1.171	0.444	0.782	1.754
Loneliness (< cut-off*)	*> cut-off*	-0.310	0.158	0.733	0.049	0.538	0.999
Self-perceived Stress	*continuous*	-0.129	0.029	0.879	**< 0.001**	0.831	0.931
Burden of exam preparation	*continuous*	-0.080	0.097	0.923	0.409	0.764	1.116
Lack of practical experience	*continuous*	-0.273	0.081	0.761	**< 0.001**	0.649	0.893

Annotation: *n* Sample size, 902, R² = 0.154, Nagelkerkes R² = 0.206. Hosmer-Lemeshow-Test: *p*= .668. Omnibus-Test: *p* < 0.001. *B* Coefficients of regression, *SE* Standard error, *OR* Odds ratio. Bold p-value= significant after Holm-Bonferroni correction within the set of comparisons reported in this table. *CI* Confidence interval. Distress = DT score > 4 points, Depression = PHQ-2 > 2 points, Anxiety = GAD-2 > 2 points, Loneliness = UCLA-3 > 5 points. (reference∗) = reference category for categorical variable

### Reported decrease in study motivation since COVID-19 pandemic

In general, 39.9% of students reported that their study motivation decreased since the COVID-19 pandemic. The share of students who affirmed this statement varied significantly by cohort, decreasing from 46.3% in winter 2021/2022 to 30.8% in summer 2023. Notably, 91 of the 155 participants in the summer 2023 cohort answered this item, as responding was optional for students for whom a pre-pandemic comparison was not applicable. Across all cohorts from winter 2021/2022 to summer 2023, students older than 25 years reported a significantly greater decrease in study motivation than their younger peers. Students in higher study years also reported a decline in study motivation significantly more frequently than students in earlier study years. In an additional post hoc subgroup analysis, clinical students reported a decrease in study motivation more frequently than pre-clinical students. After Holm–Bonferroni correction, the overall difference as well as the differences in the summer 2022 and winter 2022/2023 cohorts remained statistically significant (Supplement File 2). Moreover, the association between survey period and motivational decline remained unchanged after adjustment for age and academic year (data not shown). Regarding gender, male students reported decreased study motivation more frequently (45.8%) than female students (36.7%). On the topic of mental burden, students experiencing loneliness and distress were significantly more likely to report a decrease in study motivation compared to those unaffected by mental burdens. No significant association was observed among students with clinically relevant symptoms of anxiety and depression. Effect sizes were small to medium (Table 5). The association between survey period and motivational decline remained significant in the GEE sensitivity analysis. (Supplement File 4).

**Table 5:** Reported decrease in study motivation since COVID-19 pandemic across survey cohorts

		in total	winter 2021/ 2022	summer 2022	winter 2022/ 2023	summer 2023		
			*n* = 283	*n* = 231	*n* = 233	*n* = 91		
		%	%	%	%	*%*	χ²	*p*-value (V)
Overall decrease of study motivation since COVID-19		39.9	46.3	44.6	28.8	30.8	22.148	**< 0.001** (0.163)
Sociodemographic characteristics								
Age	*< 20 years*	21.8	37.0	12.9	15.9	16.7	34.315	**< 0.001** (0.202)
	*21–25 years*	40.9	46.1	46.9	29.0	36.0		
	*> 25 years*	51.7	54.8	56.1	47.7	35.3		
Gender	*female*	36.7	41.2	43.6	28.7	25.4	5.825 (0.016)	0.016 (0.157)
	*male*	45.8	58.8	47.0	29.0	42.9		
Study year	*Study year 1*	22.2	40.0	18.5	10.8	05.6	51.034	**< 0.001** (0.247)
	*Study year 2*	37.9	46.3	46.6	20.4	34.4		
	*Study year 3*	42.0	46.9	48.9	31.1	35.0		
	*Study year 4+*	54.8	51.9	59.7	56.7	42.9		
Study subject	*medicine*	39.3	46.1	44.4	29.4	30.1	0.016	0.899 (0.004)
	*dentistry*	38.6	55.6	41.7	69.0	37.5		
Variables of mental burden								
Depression	*< cut-off*	35.4	35.8	41.1	32.2	28.0	5.769	0.016 (0.083)
	*> cut-off*	43.5	60.3	47.5	25.4	34.1		
Anxiety	*< cut-off*	37.2	39.1	44.3	31.3	25.0	1.368	0.242 (0.040)
	*> cut-off*	41.1	55.1	44.8	27.0	35.3		
Loneliness	*< cut-off*	33.2	34.6	43.3	25.6	24.0	14.154	**< 0.001** (0.130)
	*> cut-off*	45.9	55.8	46.2	33.0	39.0		
Distress	*< cut-off*	32.7	33.7	39.0	30.4	23.3	7.120	**0.008** (0.189)
	*> cut-off*	42.4	53.3	46.5	27.9	34.4		

Annotations: *n* Sample size, % Percentage of students reporting a decrease in study motivation since the COVID-19 pandemic. χ² Chi-square statistic. *V* Cramer’s V, effect size measure for chi-square tests. Bold p-value = significant after Holm-Bonferroni correction within the set of comparisons reported in this table. Distress = DT score > 4 points, Depression = PHQ-2 > 2 points, Anxiety = GAD-2 > 2 points, Loneliness = UCLA-3 score above the established cut-off

### Medical students’ well-being

Over the course of the fading COVID-19 pandemic, mean levels of all assessed indicators of mental burden (i.e., distress, depressive symptoms, anxiety, loneliness, and perceived stress) showed varying patterns across survey waves. Loneliness generally decreased across survey waves, with the highest levels observed in winter 2021/2022 and the lowest in winter 2022/2023. In contrast, distress peaked in summer 2022, perceived stress and depression reached their highest levels in winter 2022/2023, and anxiety was highest in summer 2023. After correction for multiple comparisons, differences across survey waves remained statistically significant only for distress. Across all assessed indicators of mental burden, female students reported higher mean levels than male students, with small effect sizes (Table 6). GEE sensitivity analyses confirmed the results accounting for repeated observations. (Supplement File 4).

**Table 6:** Mental burden in 687 medical students in the fading COVID-19 pandemic in 2022 and 2023

	in total	winter 2021/ 2022	summer 2022	winter 2022/ 2023	summer 2023			
		*n* = 283	*n* = 231	*n* = 233	*n* = 155			
	*M (SD)*	*M (SD)*	*M* *(SD)*	*M (SD)*	*M (SD)*	*SQ (MS)*	*F-value*	*p-value (η²)*
Variables of mental burden								
Distress	5.93 (2.82)	5.43 (2.65)	6.42 (2.69)	5.83 (3.07)	6.29 (2.78)	138.024 (46.008)	5.875	**< 0.001** (0.019)
* > cut-off (%)*	68.3	64.3	74.5	66.1	69.7			
* in women*	6.02 (2.82)	5.52 (2.67)	6.45 (2.63)	6.02 (3.10)	6.32 (2.81)	16.145 (16.145)	2.062	0.151 (0.002)
* in men*	5.70 (2.82)	5.20 (2.62)	6.35 (2.87)	5.32 (2.94)	6.20 (2.76)			
Depression	2.58 (1.79)	2.44 (1.71)	2.64 (1.81)	2.70 (1.79)	2.59 (1.87)	2.512 (0.837)	0.262	0.852 (0.001)
* > cut-off (%)*	47.8	42.8	51.9	50.6	46.5			
* in women*	2.62 (1.78)	2.39 (1.69)	2.66 (1.81)	2.82 (1.79)	2.67 (1.86)	4.601 (4.601)	1.442	0.230 (0.002)
* in men*	2.49 (1.80)	2.56 (1.77)	2.58 (1.82)	2.37 (1.79)	2.38 (1.90)			
Anxiety	2.83 (1.83)	2.54 (1.80)	2.78 (1.79)	3.06 (1.88)	3.10 (1.78)	32.919 (10.973)	3.368	0.018 (0.011)
* > cut-off (%)*	53.9	44.9	54.1	58.8	62.6			
* in women*	2.98 (1.80)	2.66 (1.76)	2.93 (1.79)	3.26 (1.86)	3.19 (1.75)	43.887 (43.887)	13.471	**< 0.001** (0.015)
* in men*	2.45 (1.84)	2.24 (1.88)	2.41 (1.75)	2.50 (1.87)	2.85 (1.86)			
Loneliness	5.40 (2.00)	5.77 (2.02)	5.22 (1.89)	5.16 (2.00)	5.35 (1.95)	47.471 (15.824)	4.094	0.007 (0.014)
* > cut-off (%)*	47.7	55.1	45.0	42.9	45.2			
* in women*	5.51 (1.96)	5.89 (1.96)	5.32 (1.90)	5.26 (1.94)	5.48 (2.00)	27.059 (27.059)	7.001	0.008 (0.008)
* in men*	5.12 (2.02)	5.47 (2.14)	4.98 (1.88)	4.89 (2.15)	5.00 (1.77)			
Perceived Stress	6.40 (3.64)	6.30 (3.63)	6.42 (3.49)	6.48 (3.78)	6.42 (3.68)	4.141 (1.381)	0.104	0.957 (0.000)
* in women*	6.60 (3.60)	6.49 (3.61)	6.54 (3.42)	6.81 (3.75)	6.56 (3.66)	87.614 (87.614)	6.631	0.010 (0.007)
* in men*	5.87 (3.68)	5.80 (3.65)	6.12 (3.69)	5.58 (3.74)	6.03 (3.76)			

Annotations: *n* Sample size, *M* Mean value, *SD* Standard deviation, *SQ* Sum of squares, *MS* Mean square, *F* F statistic from two-way ANOVA, η² Eta-squared effect size. Bold p-value= significant after Holm-Bonferroni correction within the set of comparisons reported in this table. Distress = DT score, cut-off > 4, Depression = PHQ-2 score, cut-off > 2, Anxiety = GAD-2 score, cut-off > 2, Loneliness = UCLA-3 score, cut-off > 5, Perceived Stress = PSS-4 score. Higher scores indicate greater psychological burden

## Discussion

As medical education transitioned into the post-pandemic period of COVID-19, little was known about how medical students perceived their learning environment during this phase. This study sought to address this gap by assessing perceptions of the learning environment, study motivation, and mental well-being among German undergraduate medical students toward the end of the COVID-19 pandemic.

### Understanding medical students’ learning environment during the transition to post-pandemic medical education

Our results suggest a modest descriptive improvement in medical students’ perceptions of the MSLE following the COVID-19 pandemic, whereas other studies demonstrated greater dissatisfaction among medical students in the post-pandemic period [55, 56]. The observed differences were small in magnitude and did not remain statistically significant after correction for multiple comparisons.

The lowest proportions of students reporting a positive MSLE were observed during winter 2021/2022 and summer 2022, which coincides with pandemic-related restrictions, extensive online teaching, and increased social isolation [57]. The gradual lifting of pandemic-related restrictions and the reintroduction of in-person teaching and social interactions in academic settings may have contributed to the descriptively more positive perceptions of the MSLE in later survey waves. This pattern was accompanied by higher ratings on the JHLES-based item assessing social connectedness, highlighting that the MSLE may be shaped not only by didactic quality (e.g., in-person teaching and practical skills training) but also by students’ perceptions of social connectedness. Moreover, these findings support broader consistency across research, showing that a lack of social interaction is viewed as an important issue in medical education by both preclinical [58] and clinical students [59].

In addition, students in later survey waves reported higher levels of mentoring, potentially reflecting increased teaching and mentoring capacities among faculty members as pandemic-related restrictions eased. Hence, both structural factors - particularly in-person teaching - and relational elements, such as mentoring, appear to be central to positive perceptions of the learning environment. However, the findings should be interpreted carefully, as the survey period was not independently associated with a positive MSLE in the binary logistic regression model. Further, the attenuation of the association after adjustment for age and academic year suggests that differences in sample composition across survey waves may have partly contributed to the observed temporal variation in MSLE perceptions. Overall, findings based on the descriptive and exploratory JHLES composite score should be interpreted cautiously, as this score is based on only three selected JHLES items, does not represent a validated measure of overall MSLE, and showed low internal consistency in the present sample (Cronbach’s α = 0.474). Nevertheless, the findings regarding social connectedness and mentoring may suggest that curricular development should prioritize teaching formats that foster social interaction, such as small-group learning and mentoring.

### Differences in learning environment by students’ study year and further characteristics

Although first-year students reported the highest exploratory JHLES composite scores overall, the greatest differences in this exploratory score across survey waves were observed among students in more advanced study years. Yet, these findings should be interpreted cautiously given the exploratory score’s low internal consistency. Prior research has also noted variations in perceptions of the MSLE based on study year, with first-year students reporting overall greater MSLE satisfaction - both before [39, 52] and during the pandemic [60, 61]. However, more advanced students in this study experienced at least partial reductions in practical teaching during the pandemic. One possible explanation is that students in more advanced study years may have benefited particularly from the return to regular clinical teaching. Consistent with the importance of practical training, the binary logistic regression analysis showed that a greater perceived lack of practical experience was associated with lower odds of reporting a positive MSLE.

Our results align with prior research suggesting that supportive measures both during and after the pandemic should be tailored to different stages of medical training. Students in advanced years may particularly benefit from increased opportunities for clinical practice, including bedside teaching [62], clinical skills teaching incorporating digital formats [63], and improved academic support [64]. In contrast, undergraduate students may benefit more from additional peer-mentoring programs [64, 65], mental health support programs [66], and mindfulness-based interventions [67], which may facilitate their transition into the medical learning environment and help maintain initially high levels of motivation.

### Link of mental burden and students’ perception of learning environment

Notably, students experiencing loneliness, anxiety, depressive symptoms, or elevated distress levels reported less favorable perceptions of the MSLE. This close link between the learning environment and mental well-being is underscored by our finding that higher self-perceived stress was associated with a reduced likelihood of reporting a positive MSLE in binary regression analysis. This observation aligns with prior research conducted before COVID-19 [7, 8, 13], suggesting that the association between psychological well-being and perceptions of the learning environment is not necessarily specific to pandemic-related circumstances. However, pandemic circumstances could have exaggerated this well-known link. Thus, we are keen to emphasize that students experiencing psychological distress may particularly benefit from mental support and targeted institutional interventions, even more so in improving their MSLE.

Finally, previous research reported inconclusive findings regarding gender differences in the perception of MSLE [68–70]. In our study, the perception of the MSLE did not differ significantly by gender. Overall, this heterogeneity hints that gender may influence perceptions of MSLE, and generalizations should be made with caution.

Taken together, these findings highlight that social integration, meaningful relationships with peers and faculty members, and opportunities for mentorship are key components of a positive MSLE. As these factors remain important beyond the COVID-19 pandemic, medical curricula should continue to foster and promote them.

### The role of motivation in medical students’ academic climate

Study motivation forms another central aspect of medical education. In our study, motivation improved gradually over the study period, with the highest proportion of students reporting reduced motivation at the beginning of the study. Reduced motivation was particularly evident among those experiencing loneliness and stress. Across all cohorts, students older than 25 years and students in higher study years reported a decline in study motivation significantly more frequently than other students. One possible explanation is that students enter medical school with comparatively high levels of intrinsic and prosocial motivation and low levels of amotivation, while their expectations about the demands and sacrifices of medical training and the medical profession may initially be less realistic [71]. Consistent with this interpretation, longitudinal research has demonstrated a gradual increase in amotivation over the course of medical training [72]. The transition from preclinical to clinical training may introduce new demands like increased workload, professional socialization, perceived gaps in knowledge, and feelings of incompetence, which further challenge students’ perceptions of their learning experience as they progress through medical school [73]. These previously reported circumstances may have contributed to differences in motivational decline among students. Regarding the impact of COVID-19, this effect in more advanced students may be partially explained by disruptions to clinical training. Compared with students at the beginning of their studies, clinical students may have been more strongly affected by the disruption of patient contact and a sense of insufficient preparedness for their professional future in the rapidly changing context of the COVID-19 pandemic, similar to other research on German 4th -year students [30].

As pandemic-related restrictions eased, increased opportunities for clinical practice may have contributed to improvements in study motivation among students in more advanced study years. Nevertheless, the pandemic’s impact on study motivation does not appear uniformly negative. Other studies have reported higher levels of study motivation during the pandemic compared to pre-pandemic periods, potentially indicating adaptive responses [23, 74]. Taken together, these findings highlight the importance of supporting students who experience mental burden. Strengthening social integration and offering accessible mentoring and support programs may help to address these challenges. Regarding study motivation, male students showed a less consistent pattern across survey waves but generally reported a greater decline in motivation since the beginning of the COVID-19 pandemic. Previously, researchers have reported higher levels of amotivation among male first-year medical students [75] and male university students in general [76]. Furthermore, some found that female students tend to report higher levels of intrinsic and prosocial motivation than male students [77, 78]. Gender differences in coping strategies may have further contributed to the greater decline in motivation reported by male students [79].

However, because our study did not directly assess potential explanatory factors, our data cannot determine the underlying mechanisms of study motivation during the pandemic, which future research should explore.

### Differences in medical students’ mental well-being

The findings show that mental health burden among medical students in the late COVID-19 pandemic did not follow a uniform pattern. Loneliness was lower in later survey waves, which may reflect the gradual return of social interactions, face-to-face teaching, and extracurricular activities as pandemic-related restrictions eased.

Other indicators of mental health burden showed more complex patterns, with distress peaking in summer 2022, depressive symptoms and perceived stress reaching their highest mean levels in winter 2022/2023, and anxiety peaking in summer 2023. However, only the differences in distress between survey waves remained statistically significant after Holm-Bonferroni correction, and the observed effect sizes were small. Mean depression and anxiety scores in the present sample considerably exceeded those observed among German first-year medical students before the COVID-19 pandemic [80], as well as those reported among German medical students during the first pandemic summer in 2020 [79]. Descriptive comparisons with the general German population [47] similarly indicated higher levels of depressive and anxiety symptoms (more details are seen in Supplement File 1). Due to differences in sample composition (i.e., medical students vs. the general population), educational context (e.g., different stages of medical training), and assessment periods (e.g., varying exposure to pandemic-related restrictions), these comparisons should be interpreted cautiously. The overall high levels of mental burden may suggest that the end of pandemic-related restrictions did not necessarily result in improved mental well-being. Possible explanations include an increased workload, pressure from regularly scheduled examinations, and difficulties in readjusting to in-person teaching. Thus, difficulties in readjusting to “normal” in-person MSLE may have occurred. However, these factors were not directly assessed in our study and remain somewhat speculative. Moreover, students may have suffered long-term exhaustion from the pandemic’s prolonged challenges despite eased restrictions. Importantly, the persistently elevated levels of mental burden despite the gradual return to regular teaching suggest that factors beyond pandemic-related restrictions may contribute to psychological distress among medical students. Hence, medical schools should further consider implementing sustainable support structures to foster student well-being and resilience throughout medical training, even as pandemic-related strains ease. Consistent with previous research, female students reported higher levels of anxiety [81, 82], loneliness [83, 84], and perceived stress [85, 86].

Notably, the levels among male students also exceed those reported in other studies. However, only gender differences in anxiety remained statistically significant after correction for multiple comparisons, with a small effect size. Nevertheless, these findings support broader evidence highlighting that female medical students are particularly often subject to mental burdens. In the end, mental health concerns affect all students, and support needs to be provided to all students.

### Future perspectives: artificial intelligence and the medical learning environment

More recently, one of the most substantial changes in medical education has been the rapid introduction and widespread use of artificial intelligence (AI) and large language models (LLMs). A recent meta-analysis among medical students found that approximately three-quarters reported a positive attitude towards the use of AI, although only 36.5% felt confident in applying AI in practice [87]. AI-supported learning may positively influence the learning environment by increasing perceived engagement and reducing cognitive load [88]. Students commonly use AI to clarify complex topics, obtain individualized explanations, simplify notes, and streamline academic tasks [89]. AI may therefore facilitate knowledge acquisition, skill development, writing, and problem-solving [90]. At the same time, potential drawbacks need to be considered. Cognitive offloading and automation bias may reduce active engagement with learning material and potentially undermine intrinsic motivation and academic purpose, both important components of a positive learning environment [91]. Excessive reliance on AI may also reduce peer interaction and opportunities for collaborative learning. These concerns highlight the importance of maintaining students’ technical competencies, critical thinking, and digital literacy alongside the increasing integration of AI into medical education [90]. Nevertheless, when implemented thoughtfully, AI and LLMs have considerable potential to enhance medical education and reshape the learning environment in ways that were previously not possible.

However, because the present study did not assess the use of AI and LLMs, their potential impact on students’ perceptions of the learning environment remains beyond the scope of our findings.

## Limitations

Several limitations of the present study should be considered when interpreting the findings. First, the course and impact of the COVID-19 pandemic varied considerably across countries and regions and did not occur simultaneously worldwide. Consequently, direct comparisons between studies are complicated by differences in the timing and local development of the pandemic. In addition, the measures implemented to maintain medical education during the pandemic differed substantially both within Germany and internationally. These variations again limit the comparability of findings across various studies. This circumstance is further exacerbated by the single-center design of this study, limiting the generalizability of the findings to other medical schools and educational settings. On the other hand, this may help explain the heterogeneity of reported results, particularly regarding changes in study motivation and the inconsistent findings on preferences for online versus face-to-face teaching formats.

Another limitation lies in the sociodemographic composition of participants, particularly regarding age and academic year. Although the study also aimed to longitudinally assess medical students’ perceptions of the learning environment and mental health to investigate potential within-person changes across four distinct time points (winter 2021/2022, summer 2022, winter 2022/2023, and summer 2023), only 20 students participated at all four survey waves. Because of this limited number, more extensive longitudinal analyses were not feasible. Moreover, response rates across survey waves were relatively low, ranging from 21.1% in winter 2021/2022 to 12.0% in summer 2023. However, declining response rates have also been commonly observed in online surveys conducted during the COVID-19 pandemic [92]. Therefore, selection bias cannot be ruled out, and the generalizability of the results should be interpreted cautiously. Because of low response rates and missing longitudinal data, this study’s findings rely on cross-sectional comparisons between individual wave cohorts and do not reflect longitudinal changes within the same individuals. Furthermore, 149 participants contributed data to more than one survey wave, resulting in partially overlapping samples and non-independent observations. Although this dependence was not accounted for in the primary analyses, additional GEE sensitivity analyses incorporating within-participant correlation yielded largely consistent results. Future research should aim to ensure higher rates of repeated participation to allow for a more robust assessment of within-person changes over time.

Regarding the sample’s sociodemographic structure, this study did not assess living arrangements beyond relationship status, such as whether students lived alone or with others. Previous research has shown that medical students living alone or living far away from their families report symptoms of depression [93] and anxiety [94] more frequently than their peers. Thus, living arrangements may have acted as a potential confounding factor and should be considered when interpreting the present findings.

A methodological limitation concerns the assessment of the MSLE. Internationally, a wide range of instruments is used to evaluate learning environments in medical education. Because these instruments differ in their conceptual focus, psychometric properties, strengths, and limitations, comparisons across studies remain challenging. In this study, we assessed the MSLE using three selected items from the Johns Hopkins Learning Environment Scale and three self-developed items rather than a fully validated instrument. The exploratory composite score derived from the selected JHLES items demonstrated low internal consistency (Cronbach’s α = 0.474), limiting its reliability and the interpretability as an overall measure of the learning environment. Therefore, greater emphasis should be placed on findings from individual JHLES items, while results based on the composite score should be interpreted cautiously. Despite these methodological differences, several common trends emerge across this study, providing a meaningful basis for discussion.

Regarding the assessment of study motivation, participants may have been subject to recall bias, particularly in later survey cohorts, as students were asked to compare their current motivation with that at the beginning of the COVID-19 pandemic. Moreover, dichotomizing the ordinal scale into “decreased” and “not decreased” facilitated interpretation of the results but may have reduced information about the degree of motivational change.

## Conclusion

Our findings suggest a modest descriptive improvement in students’ global ratings of the medical school learning environment (MSLE) during the transition from mid-pandemic to post-pandemic education. These findings, together with higher ratings of peer connectedness and mentoring in later survey waves, coincided with the return of in-person teaching and clinical training in 2023. Positive perceptions of the MSLE were more frequently reported by younger students and those in earlier study years, whereas higher perceived stress and a greater perceived lack of practical experience were independently associated with lower odds of reporting a positive MSLE. Study motivation gradually increased across survey waves but remained lower in older students, students in advanced study years, and those experiencing loneliness and psychological distress. Notably, patterns of mental well-being across survey waves were less uniform following the easing of pandemic-related restrictions. While loneliness was generally lower in later survey cohorts and distress peaked in summer 2022, depressive symptoms and perceived stress reached their highest mean levels in winter 2022/2023, whereas anxiety peaked in summer 2023. Descriptive comparisons with previously published German reference samples indicated higher levels of psychological burden in the present study. Overall, these findings suggest that the gradual return to regular medical education was not necessarily accompanied by improvements in students’ mental well-being.

Taken together, our findings emphasize the importance of targeted institutional strategies that address both structural aspects of medical education, such as practical training opportunities, and relational and psychological dimensions, including stress reduction and mental health support. In particular, students in advanced stages of training and those experiencing psychological distress may benefit from tailored support measures to promote a more positive learning environment. As little is known about long-term developments in medical students’ learning environments and mental well-being following the easing of COVID-19 restrictions, continued assessment by teachers and medical faculties is warranted. More broadly, our findings suggest that promoting a positive MSLE should be regarded as a continuous priority in medical education, extending beyond periods of pandemic burden and encompassing both educational and psychosocial aspects of students’ experiences. Therefore, investing in a supportive MSLE may benefit students throughout their medical training and potentially their future professional development.

## Supplementary Information

Supplementary Material 1.

Supplementary Material 2.

Supplementary Material 3.

Supplementary Material 4.

## Data Availability

The dataset supporting the findings of this study is available from the corresponding author upon request, because ethical approval was obtained for researchers’ use only.

## Abbreviations

ANCOVA: Analysis of Covariance
ANOVA: Analysis of Variance
COVID-19: Coronavirus Disease 2019
DT: Distress Thermometer
GAD-2: Generalized Anxiety Disorder-2
JHLES: Johns Hopkins Learning Environment Scale
LE: Learning Environment
MSLE: Medical School Learning Environment
NCCN: National Comprehensive Cancer Network
OR: Odds Ratio
PHQ-2: Patient Health Questionnaire-2
PHQ-4: Patient Health Questionnaire-4
PHQ-D: Patient Health Questionnaire – German Version
PSS-4: Perceived Stress Scale-4
R-UCLA: Revised UCLA Loneliness Scale
R-UCLA-3: Three-Item Revised UCLA Loneliness Scale
SARS-CoV-2: Severe Acute Respiratory Syndrome Coronavirus 2
SPSS: Statistical Package for the Social Sciences
UMG: University Medicine Greifswald
WHO: World Health Organization
