# Supplementary material for: German medical students’ learning environment during the fading COVID-19 pandemic
Source: BMC Med Educ. 2026 Nov 11;26:1831. doi: 10.1186/s12909-026-10827-1 (PMC13753460; doi:10.1186/s12909-026-10827-1)
Supplement: Supplementary file 1 [file 12909_2026_10827_MOESM1_ESM.docx]

| **Supplementary Table 1: Descriptive comparison of depression and anxiety scores between the present sample and published German reference samples** | | | | |
| --- | --- | --- | --- | --- |
|  | **Depression** | | **Anxiety** | |
|  | **M (SD)** | **n** | **M (SD)** | **n** |
| **Present sample**  (reference) | 2.58 (1.79) | 902 | 2.83 (1.83) | 902 |
| **Students from the general German population in 2010** (Löwe et al., 2010) | 0.80 (1.18) | 176 | 0.67 (1.04) | 176 |
| **German first-year medical students pre-COVID-19** (Heinen et al., 2017) | 1.26 (1.12) | 321 | 1.40 (1.36) | 321 |
| **German medical students in summer 2020** (Guse et al., 2021) | 1.70 (1.35) | 887 | 1.43 (1.43) | 887 |
| **General German population in 2022** (Wicke et al., 2022) | 0.97 (1.21) | 4,981 | 0.83 (1.21) | 4,981 |
| Annotations: n = sample size. The present study included 902 observations from 687 individual students across four survey waves. M = mean value, SD = standard deviation. Depression = PHQ-2 sum score, Anxiety = GAD-2 sum score. Published reference data are presented for descriptive comparison only. | | | | |
