# Supplementary material for: German medical students’ learning environment during the fading COVID-19 pandemic
Source: BMC Med Educ. 2026 Nov 11;26:1831. doi: 10.1186/s12909-026-10827-1 (PMC13753460; doi:10.1186/s12909-026-10827-1)
Supplement: Supplementary file 2 [file 12909_2026_10827_MOESM2_ESM.docx]

| **Supplementary Table 2: Post hoc subgroup analyses comparing pre-clinical and clinical students** | | | | | | |
| --- | --- | --- | --- | --- | --- | --- |
|  | | *All students* | *pre-clinical*  *students* | *clinical*  *students* |  | |
|  | *n* | *%* | *%* | *%* | *χ²* | *p-value (V)* |
| **Positive perception of the Learning Environment** | | | | | | |
| **overall** | | 48.6 | 55.4 | 40.4 | 20.123 | **<.001** (0.149) |
| **winter 2021/2022** | 283 | 44.9 | 51.4 | 38.3 | 4.916 | .027 (0.132) |
| **summer 2022** | 231 | 44.2 | 52.7 | 36.1 | 6.404 | .011 (0.167) |
| **winter 2022/2023** | 233 | 54.9 | 61.7 | 46.7 | 5.279 | .022 (0.151) |
| **summer 2023** | 155 | 52.3 | 56.1 | 43.8 | 2.017 | .156 (0.114) |
| **Decrease in study motivation since the COVID-19 pandemic** | | | | | | |
| **overall** | | 39.3 | 29.9 | 49.3 | 33.033 | **<.001** (0.199) |
| **winter 2021/2022** | 283 | 46.3 | 43.0 | 49.6 | 1.273 | .259 (0.067) |
| **summer 2022** | 231 | 44.6 | 33.0 | 55.5 | 11.745 | **<.001** (0.225) |
| **winter 2022/2023** | 233 | 28.8 | 14.8 | 45.7 | 26.832 | **<.001** (0.339) |
| **summer 2023** | 91 | 30.8 | 24.0 | 39.0 | 2.387 | .122 (0.162) |
| Annotations: n= number of participants. % = percentage of students reporting an overall perception of positive Learning Environment (dichotomous) or decrease in study motivation since the COVID-19 pandemic (dichotomous). χ² = chi-square statistic. V= Cramer's V. Bold p-values = significant after Holm-Bonferroni correction within the set of comparisons reported in this table. | | | | | | |
