# Supplementary material for: German medical students’ learning environment during the fading COVID-19 pandemic
Source: BMC Med Educ. 2026 Nov 11;26:1831. doi: 10.1186/s12909-026-10827-1 (PMC13753460; doi:10.1186/s12909-026-10827-1)
Supplement: Supplementary file 3 [file 12909_2026_10827_MOESM3_ESM.docx]

# Supplementary file 3: Questionnaire

*English version of the questionnaire used in the study (German Medical Students' Learning Environment during the fading COVID-19 pandemic)*

The questionnaire comprised established measures and aswell as study-specific, self-devolped items. The items below are presented in English for supplementary publication.

**1. DT: Distress Thermometer (Ownby, 2019)**

Please circle the number (0–10) that best describes how much distress you have been experiencing in the past week, including today.

Response options:

- Continuous rating from 0 to 10

**2. PHQ-4: Patient Health Questionnaire-4 (Lowe et al., 2010)**

Over the last two weeks, how often have you been bothered by any of the following problems?

1. Little interest or pleasure in doing things
2. Feeling down, depressed, or hopeless
3. Feeling nervous, anxious, or on edge
4. Unable to stop or control worrying

Response options:

- Not at all
- Several days
- More than half the days
- Nearly every day

**3. Perceived Stress Scale – 4-item version (PSS-4) (Cohen, 1988)**

In the last month, how often have you felt that…

1. you were unable to control the important things in your life?
2. you felt confident about your ability to handle your personal problems?
3. you felt that things were going your way?
4. difficulties were piling up so high that you could not overcome them?

Response options:

- Never
- Almost never
- Sometimes
- Fairly often
- Very often

**4. Johns Hopkins Learning Environment Scale – three selected items (Shochet et al., 2015)**

1. How connected do you feel to other SOM students? (Subgroup: Community of peers)
2. Our medical school’s curriculum allows me to use my preferred learning style. (Subgroup: Academic climate)
3. I’ve found a mentor in a clinical specialty or discipline that I am passionate about. (Subgroup: Mentoring)

Response options:

- ranging from 1 = strongly disagree to 5 = strongly agree, or from 1 = not at all to 5 = tremendously

**5. UCLA 3-item Loneliness Scale (Hughes et al., 2004)**

1. How often do you feel that you lack companionship?
2. How often do you feel left out?
3. How often do you feel isolated from others?

Response options:

- Hardly ever or never
- Some of the time
- Often

**Study-specific questions, self-developed (Leich et al., 2026)**

1. How has your motivation to study changed since the beginning of the COVID-19 pandemic?

Response options:

- Significantly worsened
- Somewhat worsened
- Remained unchanged
- Somewhat improved
- Significantly improved

2. To what extent have the following aspects caused you distress during the past 4 weeks?
   a. Preparation for exams/intermediate examinations
   b. Cancellation of practical teaching (e.g., bedside teaching, clinical rotations, practical courses)

Response options:

- Not at all
- Slightly
- Strongly
- Very strongly

**References**

Cohen, S. (1988). Perceived stress in a probability sample of the United States. In S. O. Spacapan, S. (Ed.), *The social psychology of health/Sage* (pp. 31-67). Sage Publications, Inc.

Hughes, M. E., Waite, L. J., Hawkley, L. C., & Cacioppo, J. T. (2004). A Short Scale for Measuring Loneliness in Large Surveys: Results From Two Population-Based Studies. *Res Aging*, *26*(6), 655-672. <https://doi.org/10.1177/0164027504268574>

Lowe, B., Wahl, I., Rose, M., Spitzer, C., Glaesmer, H., Wingenfeld, K., Schneider, A., & Brahler, E. (2010). A 4-item measure of depression and anxiety: validation and standardization of the Patient Health Questionnaire-4 (PHQ-4) in the general population. *J Affect Disord*, *122*(1-2), 86-95. <https://doi.org/10.1016/j.jad.2009.06.019>

Ownby, K. K. (2019). Use of the Distress Thermometer in Clinical Practice. *J Adv Pract Oncol*, *10*(2), 175-179.

Shochet, R. B., Colbert-Getz, J. M., & Wright, S. M. (2015). The Johns Hopkins learning environment scale: measuring medical students' perceptions of the processes supporting professional formation. *Acad Med*, *90*(6), 810-818. <https://doi.org/10.1097/ACM.0000000000000706>
