# Supplementary material for: German medical students’ learning environment during the fading COVID-19 pandemic
Source: BMC Med Educ. 2026 Nov 11;26:1831. doi: 10.1186/s12909-026-10827-1 (PMC13753460; doi:10.1186/s12909-026-10827-1)
Supplement: Supplementary file 4 [file 12909_2026_10827_MOESM4_ESM.docx]

| **Supplementary Table 4: Sensitivity analyses accounting for repeated observations using generalized estimating equations (GEE)** | | | | | | | |
| --- | --- | --- | --- | --- | --- | --- | --- |
|  | | | | | | 95 % CI | |
| **Selected results of GEE sensitivity analyses** | | OR | B | SE | p-value | Lower CI | Upper CI |
| ***Table 4: Factors associated with positive LE - selected predictors*** | | | | | | | |
| Age | *continuous* | 0.910 | -0.094 | 0.029 | **.001** | 0.860 | 0.964 |
| Study Year (Year 1*) | *Year 3* | 0.525 | -0.645 | 0.227 | **.004** | 0.337 | 0.818 |
|  | *Year 4 +* | 0.334 | -1.097 | 0.275 | **< .001** | 0.195 | 0.573 |
| Loneliness (< cut-off*) | *> cut-off* | 0.748 | -0.291 | 0.154 | .059 | 0.553 | 1.011 |
| Self-perceived Stress | *continuous* | 0.874 | -0.135 | 0.028 | **< .001** | 0.828 | 0.923 |
| Lack of practical experience | *continuous* | 0.795 | -0.229 | 0.080 | **.004** | 0.680 | 0.931 |
| ***Table 5: Reported decrease in study motivation - survey period comparisons*** | | | | | | | |
| **Participation**  (winter 2021/2022*) | *summer 2022* | 0.922 | −0.081 | 0.167 | .629 | 0.664 | 1.280 |
|  | *winter 2022/2023* | 0.512 | −0.670 | 0.178 | **< .001** | 0.361 | 0.725 |
|  | *summer 2023* | 0.482 | −0.730 | 0.237 | **.002** | 0.303 | 0.766 |
| ***Table 6: Mental burden - survey period and gender comparisons*** | | | | | | | |
| **Distress** (winter 2021/2022*)   (female*) | *summer 2022* |  | 1.019 | 0.221 | **<.001** | 0.587 | 1.452 |
|  | *winter 2022/2023* |  | 0.383 | 0.247 | .121 | −0.102 | 0.868 |
|  | *summer 2023* |  | 0.784 | 0.254 | **.002** | 0.285 | 1.282 |
|  | *male* |  | −0.322 | 0.224 | .150 | −0.760 | 0.116 |
| **Depression** (winter 2021/2022*)  (female*) | *summer 2022* |  | 0.144 | 0.141 | .306 | −0.132 | 0.421 |
|  | *winter 2022/2023* |  | 0.163 | 0.138 | .236 | −0.107 | 0.433 |
|  | *summer 2023* |  | 0.026 | 0.166 | .878 | −0.300 | 0.351 |
|  | *male* |  | −0.103 | 0.147 | .486 | −0.391 | 0.186 |
| **Anxiety** (winter 2021/2022*)   (female*) | *summer 2022* |  | 0.246 | 0.143 | .085 | −0.034 | 0.527 |
|  | *winter 2022/2023* |  | 0.453 | 0.150 | **.003** | 0.159 | 0.747 |
|  | *summer 2023* |  | 0.493 | 0.163 | **.003** | 0.173 | 0.813 |
|  | *male* |  | −0.501 | 0.149 | **< .001** | −0.793 | −0.209 |
| **Significant variables in primary analyses (continuation)** | |  | B | SE | p-value | Lower CI | Upper CI |
| **Loneliness** (winter 2021/2022*)   (female*) | *summer 2022* |  | −0.533 | 0.151 | **< .001** | −0.829 | −0.238 |
|  | *winter 2022/2023* |  | −0.604 | 0.162 | **< .001** | −0.922 | −0.286 |
|  | *summer 2023* |  | −0.384 | 0.178 | .031 | −0.733 | −0.036 |
|  | *male* |  | −0.426 | 0.164 | .009 | −0.747 | −0.105 |
| **Perceived Stress** (winter 2021/2022*)   (female*) | *summer 2022* |  | 0.048 | 0.273 | .861 | −0.488 | 0.583 |
|  | *winter 2022/2023* |  | −0.057 | 0.277 | .836 | −0.599 | 0.485 |
|  | *summer 2023* |  | −0.093 | 0.309 | .762 | −0.698 | 0.511 |
|  | *male* |  | −0.757 | 0.300 | .012 | −1.345 | −0.169 |
| Annotations: n = sample size (Table 4: 902; Table 5: 838; Table 6: 902). GEE = generalized estimating equations. B = coeﬃcients of regression, SE = standard error, OR = Odds ratio, CI = confidence interval for OR in table 4, and table 5; coefficients of regression in table 6. Bold p-value= significant after Holm-Bonferroni correction within the set of comparisons reported in this table (i.e., table 4, table 5, and table 6). (reference∗) = reference category for categorical variable. Variables in Table 4: Loneliness = UCLA-3 score, cut-off > 5, Variables in Table 6: Distress = DT sum score, Depression = PHQ-2 sum score, Anxiety = GAD-2 sum score, Loneliness = UCLA-3 sum score, Perceived Stress = PSS-4 sum score. Higher scores indicate greater psychological burden. | | | | | | | |
